# p57^Kip2^ Phosphorylation Modulates Its Localization, Stability, and Interactions

**DOI:** 10.3390/ijms252011176

**Published:** 2024-10-17

**Authors:** Emanuela Stampone, Debora Bencivenga, Luisa Dassi, Sara Sarnelli, Luisa Campagnolo, Valentina Lacconi, Fulvio Della Ragione, Adriana Borriello

**Affiliations:** 1 Department of Precision Medicine, University of Campania “Luigi Vanvitelli”, 80138 Naples, Italy; debora.bencivenga@unicampania.it (D.B.); luisa.dassi@unicampania.it (L.D.); sara.sarnelli@unicampania.it (S.S.); fulvio.dellaragione@unicampania.it (F.D.R.); 2 Department of Biomedicine and Prevention, University of Rome Tor Vergata, 00133 Rome, Italyvalentina.lacconi@uniroma2.it (V.L.)

**Keywords:** CIP/Kip, p57^Kip2^, phosphorylation, PTM, two-dimensional analysis, cell cycle

## Abstract

p57^Kip2^ is a member of the cyclin-dependent kinase (CDK) Interacting Protein/Kinase Inhibitory Protein (CIP/Kip) family that also includes p21^Cip1/WAF1^ and p27^Kip1^. Different from its siblings, few data are available about the p57^Kip2^ protein, especially in humans. Structurally, p57^Kip2^ is an intrinsically unstructured protein, a characteristic that confers functional flexibility with multiple transient interactions influencing the metabolism and roles of the protein. Being an IUP, its localization, stability, and binding to functional partners might be strongly modulated by post-translational modifications, especially phosphorylation. In this work, we investigated by two-dimensional analysis the phosphorylation pattern of p57^Kip2^ in different cellular models, revealing how the human protein appears to be extensively phosphorylated, compared to p21^Cip1/WAF1^ and p27^Kip1^. We further observed clear differences in the phosphoisoforms distributed in the cytosolic and nuclear compartments in asynchronous and synchronized cells. Particularly, the unmodified form is detectable only in the nucleus, while the more acidic forms are present in the cytoplasm. Most importantly, we found that the phosphorylation state of p57^Kip2^ influences the binding with some p57^Kip2^ partners, such as CDKs, LIMK1 and CRM1. Thus, it is necessary to completely identify the phosphorylated residues of the protein to fully unravel the roles of this CIP/Kip protein, which are still partially identified.

## 1. Introduction

p57^Kip2^ (hereinafter p57) is a cyclin-dependent kinase inhibitor (CKI) belonging to the cyclin-dependent kinase (CDK) Interacting Protein/Kinase Inhibitory Protein (CIP/Kip) family, which also includes p21^Cip1/WAF1^ and p27^Kip1^ [1,2]. p57 is the least studied family member and, for a long time, its relevance has been associated with its central role during embryogenesis and development [3]. Indeed, unlike p21^Cip1/WAF1^ and p27^Kip1^, *Cdkn1c* (the mouse p57 encoding gene) knock-out mice showed high neonatal mortality and serious organ defects during development and growth [4,5,6]. In addition, genetic studies on patients with Beckwith–Wiedemann syndrome (BWS), characterized by overgrowth, a predisposition to tumors, and congenital malformations, showed that alterations in the *CDKN1C* coding sequence have functional significance in the pathogenesis of the syndrome [7,8,9]. Subsequently, *CDKN1C* mutations were found to be involved in the pathogenesis of two other growth-related syndromes, such as IMAGe (Intrauterine growth restriction, Metaphyseal dysplasia, Adrenal hypoplasia congenita, and Genital anomalies) and Russell–Silver syndrome (RSS), consolidating the idea of the key role of p57 during embryonic development and in the homeostasis of tissues/organs expressing the protein [10,11,12,13]. Since the loss of p57 expression has pathological relevance, most of the p57 information is related to the molecular mechanisms that regulate its expression level [14,15,16,17]. Conversely, few data are reported about the protein, including its half-life, mechanisms of removal, post-translational modifications (PTMs), and interactors.

Identified by homology with p27^Kip1^, human p57 is the largest protein of the family, consisting of 316 amino acids with a theoretical molecular weight of about 32 kDa and an apparent molecular weight in SDS-PAGE of 57 kDa [1,2]. The remarkable difference between the molecular weight calculated on the sequence basis and that estimated by SDS-PAGE is probably due to the presence of a characteristic central domain called the “PAPA region” made of Pro/Ala repeats, which are not present in the other CIP/Kip proteins [2,18]. It has been proposed that this region is necessary for p57 interaction with LIMK1 (LIN-11, Isl-1, MEC-3 kinase 1), a Ser/Thr kinase involved in the regulation of actin fibers. All the CIP/Kip proteins participate in the control of actin dynamics, modulating the activity of different effectors of the Ras Homolog Family Member A (RhoA)/Rho associated coiled-coil containing protein kinase (ROCK)/LIMK1 signaling [19]. LIMK1 is the downstream effector of the RhoA/ROCK pathway, and, when activated, it phosphorylates and inhibits the actin-severing protein cofilin, stabilizing the actin filament [20]. It has been reported that the p57-LIMK1 activating interaction in the cytoplasm of several cancer cell lines determines actin stabilization and stress fibers’ formation, with the consequent inhibition of cancer cell migration and/or the induction of apoptosis [21,22,23,24,25]. Conversely, p57 shares with p21^Cip1/WAF1^ and p27^Kip1^ a highly conserved region at the N-terminus, called the Kinase Inhibitory Domain (KID), which appears to be necessary and sufficient to bind and inhibit the cyclin/CDK complexes mainly (but not exclusively) at the G1/S transition, i.e., the complexes Cyclin Ds/CDK4(6) and Cyclin E(A)/CDK2 [26,27]. At the protein C-terminus, the Proliferating Cell Nuclear Antigen (PCNA) binding domain, homologous to that of p21^Cip1/WAF1^ [28], and the QT (Glutamine Threonine) domain, homologous to that of p27^Kip1^, are recognizable [1,2]. Although a clear definition of the QT domain function has yet to be determined, the importance of the PCNA binding domain is well documented. PCNA is a co-factor of the DNA polymerase delta and epsilon, which recruits other players at the replication fork to enhance DNA replication or to allow DNA repair [29]. The strong binding of p21^Cip1/WAF1^ to PCNA displaces DNA polymerases, resulting in DNA replication inhibition [29]. Since the PCNA binding domain of p21^Cip1/WAF1^ overlaps the binding region to the C8α-20S subunit of the proteasome, it has been demonstrated that the association with PCNA protects p21^Cip1/WAF1^ from degradation [30,31,32]. Similarly, the PCNA-binding domain of p57 is necessary for the stability of the protein despite showing a much lower binding affinity. Most of the information on the p57 C-terminal domain comes from the analysis of *CDKN1C* mutations found in probands with IMAGe and RSS, two hypoproliferative growth-related syndromes with some shared clinical features [10,11,12,13]. Specifically, these germinal mutations, disrupting the PCNA binding domain of p57, lead to an increased stability of the protein, with the consequent impairment of proliferation and altered embryonal development [10,33]. Finally, a bipartite nuclear localization signal (NLS) is present at the C-terminal moiety of p57. Specifically, we recently identified a new NLS at the last five amino acids of the protein sequence while investigating the effect of a BWS-associated missense mutation (namely, Arg316Trp, c.946 C>T) on p57 cellular localization [34]. The finding allowed us and others to characterize the presence of a bipartite NLS at the p57 C-terminus, consisting of two basic Arg-Lys motifs, with the first (278-KRKR-281) identified by homology with the mouse p57 and with other CIP/Kip proteins, and the second at residues 312-RKRLR-316 [34,35].

p57 is an Intrinsically Unstructured Protein (IUP), a characteristic that confers high conformational flexibility [36]. In turn, this feature allows IUPs to adapt to numerous different interactors. In this scenario, IUP PTMs are of peculiar relevance, playing a critical role in modulating plasticity, interactors, and functions [37]. Thus, many functions of IUPs may be switched “on” or “off” by conformational changes induced by PTMs, particularly by phosphorylation [37]. Accordingly, there is an extensive amount of the literature documenting how phosphorylations influence the metabolism and functions of p21^Cip1/WAF1^ and p27^Kip1^ [38,39,40], while very few studies are available about the PTMs of human p57 [14,18,41].

It is known that mitogenic signals activate intracellular signaling pathways that induce the nuclear removal of CKIs and/or their cytoplasmic delocalization to allow cell cycle progression [42]. As a matter of fact, the phosphorylation of target proteins has a key role in driving them to degradation via the ubiquitin–proteasome system [43]. Some studies have suggested that the p57 cellular levels are modulated by the ubiquitin–proteasome pathway [42,44,45]. Particularly, it was found that Skp2 (S-phase kinase associated protein 2), the well-known F-box protein acting as substrate recognition in the Skp1/Cul1/F-box protein (SCF) ubiquitin ligase complex, is also involved (like for p27^Kip1^ and p21^Cip1/WAF1^) in the recognition of p57 [42]. In analogy with p27^Kip1^, it has been reported that Skp2 interacts with p57 and promotes its degradation when the CKI is phosphorylated on a Threonine residue present at the C-term of the protein, particularly on Thr 310 [44]. Human p57 mutated on Thr 310 was not recognized by Skp2, suggesting that phosphorylation on this residue creates a phosphodegron addressing the protein to proteasome degradation by the SCF-Skp2 complex [46,47]. Additionally, in breast cancer cell lines, also the phosphorylation at Ser 282 has been associated with p57 proteasomal-dependent degradation [48]. In more detail, in HER2-overexpressing cancer cell lines, it has been shown that the serine/threonine kinase Akt is capable of phosphorylating p57 at Ser 282 or Thr 310, causing the cytoplasmic delocalization of the protein and proteasomal degradation, and highlighting that Akt activity results in the destabilization of p57 by accelerating its turn-over rate [48]. In mice, beside the SCF-Skp2 ubiquitin ligase complex, another F-box protein, the F-box Leucine-rich repeat protein 12 (FBL12), was reported to be responsible for p57 recognition by the ubiquitin-dependent proteasomal degradation system [45,49,50,51]. In murine osteoblast cells, TGF-β1 (Transforming Growth Factor beta 1) stimulates the Smad-dependent transcription of the gene encoding FBL12, allowing p57 proteolysis. Based on the homology with Thr 310 in human p57, the authors showed that the SCF-FBL12 complex binds to and ubiquitylates mouse p57 upon the recognition of phosho-Thr 329 phosphodegron [45,49,50,51]. Of interest, the TGF-β1 stimulation of osteoblast cells does not influence the ubiquitylation and proteasomal degradation of the other two CIP/Kip proteins p21^Cip1/Waf1^ and p27^Kip1^, highlighting peculiarities of p57 regulation during tissue differentiation [45,50,51].

In addition to protein degradation, cytoplasmic translocation contributes to the reduction in the nuclear content of CIP/Kip proteins. Particularly, in the early G1 phase, phosphorylation at Ser 10 of p27^Kip1^ favors the binding to the major exporter protein CRM1 (Chromosome Region Maintenance 1/exportin1), determining the rapid export of pSer10-p27 from the nucleus [52,53,54]. However, whether the interaction with CRM1 also determines the cytoplasmic translocation of p57, and whether the binding is influenced by phosphorylation still remains elusive. In mice, it has been reported that the nuclear-cytoplasmic shuttling of p57 is required for the differentiation of oligodendrocytes [55]. Moreover, the selective inhibition of CRM1 activity prevented the cytoplasmic translocation of p57 and impaired differentiation [55]. In Her2-positive breast cancer cell lines, p57 phosphorylation at Ser 282 has also been associated with the cytoplasmic delocalization of the protein and with the increased proteasomal-dependent degradation [48].

Mitogenic signals can activate non-receptor tyrosine kinases, such as the proto-oncogene products Src and Abl. It has been proposed that Src might phosphorylate p57, similarly to p27^Kip1^. Even though the mechanism has not been elucidated, the authors proposed a role for Src in promoting p57 degradation through phosphorylation and subsequent ubiquitylation [56]. It is known that the tyrosine phosphorylation of CIP/Kip proteins can influence their binding with CDKs [57]. Regarding p27^Kip1^, phosphorylation at tyrosines 74, 88, and 89 has been involved in the differential modulation of CDKs’ activity, mostly acting in a context-dependent manner [58,59]. Human p57 has two tyrosyl residues, Tyr 63 and 91, but their involvement in modulating CDK binding has not yet been investigated. Similarly, no experimental evidence has been reported on how phosphorylation can modulate the binding of p57 to other functional partners in both the nuclear and cytosolic compartments.

In a previous study, we observed that p57 is abundantly phosphorylated in the chronic myelogenous leukemia cell line K562 [60]. Considering the relevance of the phosphorylation reported for the other CIP/Kip proteins, it appears crucial to characterize the p57 phosphorylations and to understand their functions. To this purpose, in this work, we characterized the phosphorylation pattern of the protein in different cell models, as well as in asynchronous and synchronized cells. We discovered that p57 occurs mostly as a phosphorylated protein and that the amount of the unmodified protein represents a minor percentage of the total p57 content. In addition, we investigated how the phosphorylation influences the stability, localization, and binding of the protein with known interactors, like CDKs, LIMK1, and CRM1.

## 2. Results

### 2.1. Characterization of the p57 Isoform Pattern

**Figure 1:**
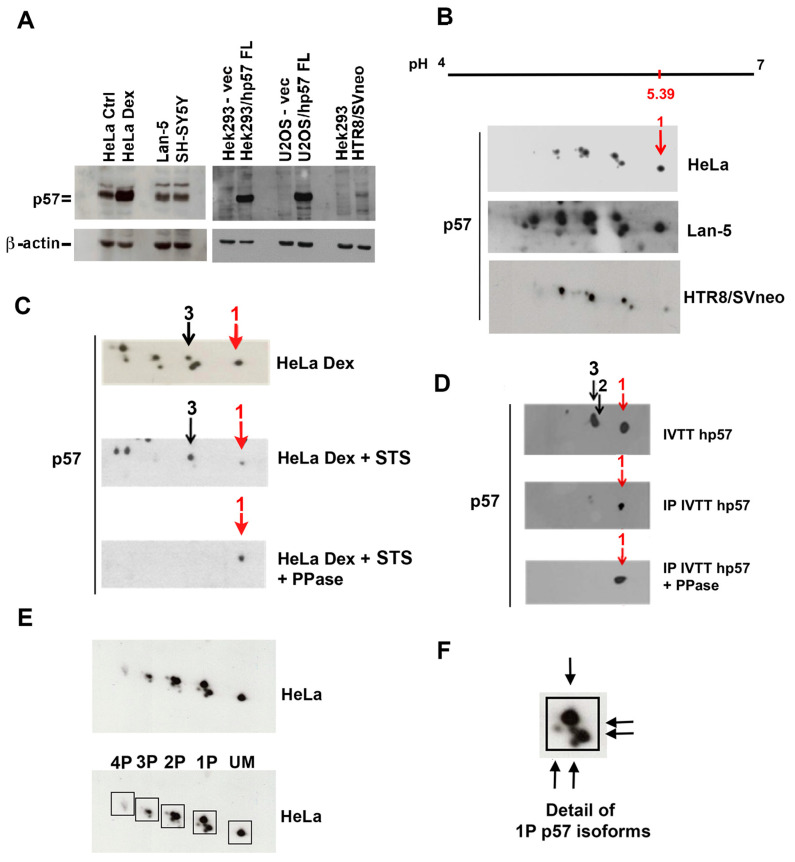
Characterization of the p57 isoform pattern. (**A**) 1D/WB analysis of p57 content in different cell lines, like HeLa ctrl and HeLa treated with 100 nM of Dex for 24 h to increase p57 levels, the neuroblastoma cell lines Lan-5 and SHSY5Y, the trophoblast cell line HTR8/SVneo, and Hek293 and U2OS cells transfected with the hp57-FL plasmid. Loading was verified by *β*-actin analysis (**B**) 2D/WB of p57 from total protein extracts of HeLa, Lan-5 and HTR8/SVneo cells. The linear range of pH 4–7 was employed for the isoelectrofocusing, since the pI of the FL-hp57 is 5.39. Accordingly, the spot focusing at the reported pH is indicated with number 1 (red arrow). (**C**) The 2D/WB analysis of p57 in HeLa cells treated as indicated. Cells were grown in medium supplemented with 100 nM of Dex for 24 h. Subsequently, Dex-treated cells were incubated with staurosporine (STS), a broad-spectrum kinase inhibitor. Then, cells were collected and lysed. Half of the total protein extract was incubated with λ-PPase and then analyzed by 2D/WB in comparison with the PPase-untreated extract and the extract from DEX-exposed cells. Spots from 1 to 3 were indicated by arrows (red arrow indicates spot 1). Mainly, spots 1 and 3 were maintained after staurosporine treatment, then spot 3 disappeared upon PPase assay. (**D**) The 2D/WB analysis of recombinant hp57-FL (hp57) was produced by the IVTT reaction. Arrows indicate the unmodified form (spot 1, red arrow) and two monophosphorylated isoforms (spots 2 and 3). Recombinant hp57 was immunoprecipitated with a rabbit pAbs anti-p57 to confirm the specificity of the detected p57 signal and then incubated with λ-PPase. The 2D/WB analysis showed only the spot focused at pH 5.39 (in red), corresponding to the unmodified hp57. (**E**) The 2D/WB analysis of p57 isoforms in HeLa cells. hp57 isoforms that focus at the same pH are grouped in boxes. The isoform that focuses at pH 5.39 corresponds to the unmodified form of p57 (UM), while shifting toward the acidic pole (left) indicates forms to which a phosphate group is progressively added. 1P, 2P, 3P, and 4P indicate hp57 isoforms containing, respectively, one, two, three, and four phosphate groups. (**F**) The image shows a detail of panel (**E**). A magnification of hp57 monophosphorylated (1P) forms is shown. Arrows point to five distinct monophosphorylated isoforms detected in HeLa cells.

We have previously showed that the mono-dimensional (1D) immunoreactive signal of p57 appears as a doublet [22,44]. We also discovered, by two-dimensional Western blotting (2D/WB), that in K562 cells, p57 presents numerous distinct phosphoisoforms [60]. To confirm and extend these previous findings, the 2D/WB isoform pattern of p57 was analyzed in additional cell models. The cellular content of p57 was first verified by 1D/WB in two neuroblastoma cell lines, Lan-5 and SH-SY5Y, in the cervical cancer cell line HeLa treated (or not) for 24 h with 100 nM Dexamethasone (Dex), which is known to increase the p57 level, and in non-tumoral cells such as the HTR8/SVneo trophoblast cell line. As a control, the protein extracts of two cell lines, Hek293 (non-tumoral human embryonal kidney cell line) and U2OS (established from human osteosarcoma), respectively, transfected with an empty pcDNA3.1 plasmid and a vector containing the coding DNA sequence (CDS) for the full-length (FL) human p57 were used. Hek293 and U2OS cells were selected to overexpress p57 since they had undetectable levels of the protein, according to Western blot analysis (Figure 1A).

As shown in Figure 1A, the immunoreactive signal of p57 appeared as a doublet in the analyzed extracts, suggesting the presence of PTMs of the protein. To confirm this hypothesis, the protein cell extracts were subsequently analyzed by 2D/WB.

Figure 1B shows that the p57 isoform pattern is highly conserved among cell lines, with numerous (at least eight) clearly distinct immunoreactive spots. Based on the theoretical isoelectric point of the native protein (pI, pH 5.39), spot 1 has been associated with the unmodified p57 protein, whereas more acidic spots correspond to putatively different post-synthetically modified isoforms. We also postulated that most of the spots correspond to differently phosphorylated p57 isoforms. To confirm the hypothesis, we employed two distinct strategies, one based on the characterization of p57 from cell extracts treated with a kinase inhibitor and the other based on an in vitro transcription and translation (IVTT) system. Accordingly, HeLa cells were incubated with 100 nM Dex for 5 h to increase the amount of the protein (as in Figure 1A). After one hour, the cells were also exposed to 5 µM staurosporine (STS), a broad-spectrum kinase inhibitor. Then, cells were collected and lysed for protein extraction and subsequent 2D/WB analysis. As reported in Figure 1C, STS treatment reduces the Dex-dependent p57 up-regulation and inhibits the appearance of most of the p57 immunoreactive spots. In addition, the remaining p57 signals were converted into spot 1 upon the subsequent incubation of STS-treated cell extracts with λ-PPase (Figure 1C). The result suggested that spot 1 corresponds to the unmodified native form of p57, and that all the other spots are most likely phosphoisoforms. In addition, recombinant human p57 (hp57) was prepared by an IVTT reaction employing the hp57-pcDNA3.1 plasmid. The assay mixture was then analyzed by 2D/WB. As shown in Figure 1D, three main signals were evidenced, marked as spots 1, 2, and 3. Subsequently, IVTT hp57 was immunoprecipitated, incubated with λ-PPase and analyzed by 2D/WB. Only one signal was observed, which focused at the same pH of spot 1, confirming the identification of spot 1 as the unmodified hp57 (Figure 1D). To simplify the description of the complex p57 2D pattern, we grouped the isoforms of p57 (from the HeLa cellular extract, as an example) on the basis of the pI shift calculated by adding one (or more) phosphate to the protein. By this method, we obtained the image reported in Figure 1E. Similar p57 2D patterns were observed in Hek293 (embryonal non-tumoral) and U2OS (tumoral) cell lines transfected with the hp57-pcDNA3.1 plasmid (Figure S1). The enlarged analysis of monophosphorylated isoforms, reported in Figure 1F, showed at least five spots. The finding allowed us to hypothesize the occurrence of at least five distinct phosphorylatable residues in p57.

In summary, 2D/WB analyses demonstrated that the phosphorylated p57 isoforms represent the large majority of the p57 cellular content. We concluded that human p57 must be considered as a phosphoprotein and that phosphorylation might be of critical relevance in p57 functions, such as the modulation of the activity of cyclin/CDK complexes through cell cycle phases, the regulation of cell differentiation, and the stress response. Indeed, for example, the phosphorylation pattern of p57 varies in appropriately differentiated (Figure S2) and/or stressed cells (Figure S3), implying that various p57 isoforms perform specific roles.

Then, we focused our interest on the role of p57 phosphorylation in protein interaction and localization.

### 2.2. Compartmentalization of p57 Phosphoisoforms

**Figure 2:**
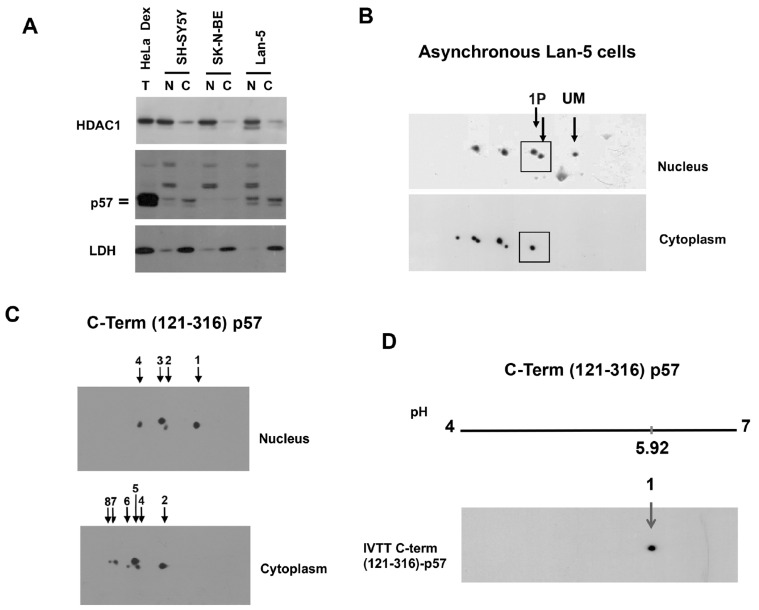
Analysis of p57 isoforms in the cytoplasmic and nuclear compartments. (**A**) The 1D/WB analysis of p57 in nuclear and cytoplasm fractionation of the neuroblastoma cells SH-SY5Y, SK-N-BE, and Lan-5. Total extract of HeLa treated with Dex was used as a reference for the hp57 signal. HDAC1 and LDH were evaluated as markers of nuclear and cytosolic fractions, respectively. (**B**) The 2D/WB of p57 in the nuclear and cytoplasmic fractions of asynchronous Lan-5 cells. Arrows indicate the unmodified (UM) p57 form detected only in the nucleus and the other two monophosphorylated (1P) forms. A box is also added to group the 1P p57 isoforms, in nuclear and cytoplasmic fractions. (**C**) The 2D/WB of the nuclear and cytoplasmic fractions of HeK293 cells transfected with pcDNA3.1 plasmid expressing the C-term (121–316)-p57 fragment. Eight isoforms of the C-term fragment were detected and numbered from the basic to acidic pH. Spot 1 (unmodified C-term fragment) was detected only in the nucleus. The less phosphorylated forms of the fragment were detected in the nucleus (spots 2–4), while the most phosphorylated forms localized in the cytoplasm. (**D**) The 2D/WB of the IVTT reaction of the C-term (121–316)-p57 fragment to confirm that spot 1 corresponds to the unmodified form of the fragment. Only one spot was detected that focused at pH 5.92, corresponding to the theoretical calculated pI of the C-term fragment.

p57 is an IUP. PTMs, including phosphorylation, are considered crucial for modulating the subcellular localization, stability, interactors, and functions of the IUPs. Initially, we characterized p57 phosphoisoforms in different cellular compartments. Cytoplasmic and nuclear extracts from three asynchronous neuroblastoma cell lines were prepared and analyzed by 1D/WB (Figure 2A). SK-N-BE cells showed a very low expression of p57 in both compartments. Although p57 is mostly considered a nuclear protein, it appears to be clearly detectable in the cytoplasm of SH-SY5Y and Lan-5 cells, at levels comparable, if not higher, than those in the nucleus. Additionally, the immunoreactive doublet is evident in both subcellular fractions, suggesting the occurrence of p57 phosphorylation either in the nuclear or cytosolic compartments. To characterize the differential distribution of p57 isoforms, cytoplasmic and nuclear extracts from asynchronous Lan-5 cells were analyzed by 2D/WB. In Figure 2B, a clear separation of the p57 isoforms was evident. Particularly, the unmodified form (UM) was present only in the nuclear extract of the asynchronous cells, while the phosphorylated forms were particularly abundant in the cytoplasm. Nonetheless, one of the two monophosphoforms (in the 1P square) colocalizes with the UM only in the nuclear preparation. The absence of the unmodified form in the cytoplasm allowed us to hypothesize that the protein phosphorylation(s) status might modulate nuclear exports/imports and/or that the different phosphoisoforms specifically control distinct processes in the two cellular compartments.

To confirm the occurrence of unmodified p57 only in the nuclear compartment, we transfected a p57 expression vector codifying for the last 196 residues of the protein, (121–316)-p57. This C-term p57 fragment includes the bipartite NLS [34] and, thus, it should be able to enter the nucleus. After transfection, we separated the nuclear and cytosolic compartments and analyzed the extract for the p57 fragment by 2D/WB. As reported in Figure 2C, the two compartments showed clearly distinct isoform patterns, with the spot that, according to the pI, corresponds to the unmodified form (spot 1) being present only in the nucleus. The cytosol, conversely, showed the occurrence of multiple phosphorylated isoforms. A difference is also evident in the distribution of the 1P forms. Particularly, spot 3 localizes only in the nucleus. To confirm that spot 1 corresponds to the unmodified form of the (121–316)-p57, the recombinant C-term p57 fragment was prepared by an IVTT reaction as performed for the FL-hp57 and analyzed by 2D/WB. In Figure 2D, only one spot was detected that focalizes at pH 5.92. This value corresponds to the pH at which spot 1 focalizes in the 2D pattern of the C-term (121–316) p57 (Figure 2C). The experiment confirms our characterization of spot 1 as UM, and spots 2 to 8 as phosphoforms. Also, the compartmentalization analysis confirms that the unmodified form and one specific monophosphoisoform (spot 3) of the C-term fragment localize in the nucleus and are not detected in the cytosol.

### 2.3. Cell Cycle-Dependent Modulation of p57 Phosphorylation(s) and Evaluation of Phosphoisoforms Involved in the CDKs’ Interaction

**Figure 3:**
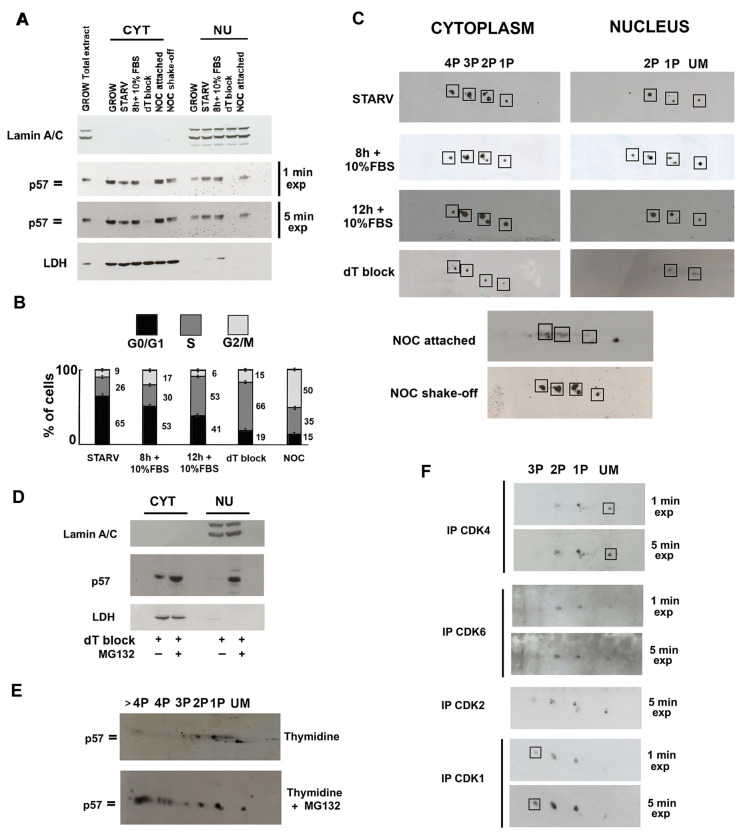
Analysis of p57 isoforms in synchronized cells and evaluation of the binding with CDKs. (**A**) 1D/WB analysis of p57 content in nuclear and cytoplasm fractionation of synchronized Lan-5 neuroblastoma cells obtained through the reported treatment: starvation (STARV) for enrichment in G0/G1; addition of 10% FBS to starved cells (+10% FBS for 8 and 12 h) for cells entering G1 and proceeding toward the S phase; thymidine block for the S phase arrest (dT block); harvesting of attached and shaking-off detached cells after nocodazole treatment (for the G2 and M phase, respectively). Total extract from unsynchronized (GROWING) cells was used as a reference. Western blotting of Lamin A/C and LDH was employed as a marker of, respectively, nuclear and cytoplasmic fractions. (**B**) Graphical representation of FACS analysis results related to Lan-5 cell synchronizations performed as described in the Material and Methods. (**C**) The 2D/WB analysis of hp57 in the nuclear and cytoplasmic fractions of synchronized Lan-5 cells shows the distribution pattern of p57 phosphoisoforms in cell cycle phases. As in Figure 1E, hp57 isoforms that focus at the same pH are grouped in boxes. The unmodified (UM) and the mono- and biphosphorylated forms (1P and 2P) of hp57 forms are detected in the nucleus through cell cycle phases, while the most phosphorylated hp57 forms (from 1P to 4P) are detected in the cytoplasm in each analyzed phase of the cell cycle (except the S phase). In the G2/M phase, it is evident that hp57 is highly phosphorylated. (**D**) The 1D/WB of cytoplasmic and nuclear fractionation of Lan-5 arrested in the S phase after 48 h of incubation with 2 mM deoxythymidine (dT block). A total of 10 µM of MG132 was added in the last 5 h of dT incubation to prevent proteasome-dependent protein degradation. As in panel A, Western blotting of Lamin A/C and LDH were employed as a marker of nuclear and cytoplasmic fractions, respectively. (**E**) The 2D/WB analysis of hp57 in total Lan-5 extracts arrested in the S phase treated or not with MG132, as in panel (D). (**F**) The 2D/WB analysis of p57 isoforms involved in the binding with CDK4, CDK6, CDK2, and CDK1. A total of 5 mg of Lan-5 extract was used for immunoprecipitation of each CDK. Images acquired at different blot exposure times (1 min and 5 min of exposure) are reported. Boxes highlight the main p57 forms involved in the binding.

It has been definitely established that the phosphorylation of CIP/Kip proteins controls both their localization and stability in a cell cycle phase-dependent manner. Thus, the subcellular distribution of p57 isoforms was examined in synchronized cells. Accordingly, Lan-5 cells were synchronized in G0 (starved cells), and then allowed to re-enter the cycle from starvation (in G1 toward S) by incubation in medium added with 10% FBS. Cells were collected for subsequent analysis after 8 h and 12 h of incubation. Other treatments include a thymidine block to arrest cells in the S phase, and a nocodazole treatment followed by a separate harvesting of attached cells and shaking-off detached cells for obtaining cells in G2, and the M phase, respectively, as fully described under the “Materials and Methods” section. Nuclear and cytoplasmic extracts were prepared and analyzed by 1D/WB and 2D/WB. No intact nuclei were obtained from detached cells upon nocodazole exposure, as expected for cells in mitosis. As shown in the 1D/WB in Figure 3A, p57 cellular content did not change significantly through cell cycle progression, except for the S-phase, in which p57 levels dropped dramatically in both compartments. In the S-phase, p57 levels rapidly decreased, as also observed for the other two CIP/Kip proteins. The phosphorylation status of p57 strongly changed through the cell cycle phases, highlighting clear differences in the immunoreactive signals of p57 between the two compartments. In particular, in the nucleus, a doublet was detected constantly. Conversely, in the cytosol, p57 appeared mainly as the faster migrating immunoreactive band, while the progressive appearance and accumulation of the upper/slower band was observed from G1 to the M (excluding S) phase. The finding demonstrates the cell cycle-dependent modulation of the phosphorylation pattern of p57. Moreover, the result allowed us to hypothesize that some phosphoisoforms, comigrating in the upper band (slower migrating band) of the immunoreactive p57 signal, can be involved in the export of the protein from the nucleus in a cell cycle phase-dependent manner. This view is suggested by the trend in the slower migrating band, constantly present in the nucleus and gradually accumulating in the cytoplasm as the cell cycle proceeds.

Then, the 2D/WB phosphoisoform patterns of cytosolic and nuclear p57 at various stages of the cell cycle were investigated. Figure 3B reports the FACS analysis confirming the synchronization. No sub-G1 peak was observed in any analysis. For the G2/M phase, total cellular extracts were analyzed. As shown in Figure 3C, distinct patterns of p57 phosphoisoforms were detected. In accordance with the data in Figure 2B, the more acidic forms of the protein were present in the cytoplasm, while the less-modified forms appeared in the nucleus. Moreover, in the G2/M phase (attached and shaken-off cells upon the nocodazole), the pattern shifted toward even more acidic isoforms. In detail, in the cytoplasm of the G1/S phase, the number of further forms with a slower electrophoretic speed increased. Accordingly, the inversion of the relative intensities of the spots comprised in the 2P group was observed as the cell cycle proceeded (from the G0 to the G1/S phase). A similar progressive change was detected in the 1P group in the nuclei, where the increasing intensity of the upper spot was evident from the G0-to-G1/S transition, together with the reduction in the nuclear content of the protein. This suggested that this phosphoisoform might play a role in modulating the stability of the nuclear p57 at the G1/S transition of the cell cycle. To verify this hypothesis, S-phase-arrested cells were treated with 10 µM of proteasome inhibitor MG132 for 5 h and subsequently analyzed by 1D/WB and 2D/WB. As shown in Figure 3D, in Lan-5 cells arrested in S-phase, p57 was not visible in the nuclear extract but was still detectable in the cytoplasm. This evidence confirmed results reported in Figure 3A (5 min film exposure). The treatment with MG132 was effective in determining the accumulation of the protein both in the nucleus and cytoplasm. In Figure 3E, the 2D/WB analysis of the nuclear extracts from S-phase-arrested (by thymidine block) Lan-5 cells treated or not with MG132 showed the increased intensity of the 1P upper isoform, together with the accumulation of more acidic isoforms not detected before. This result confirms our hypothesis that the upper monophosphorylated form is involved in the degradation of p57 via the ubiquitin–proteasome system. It has been reported that phosphorylation at Ser 282 or at Thr 310 causes the proteasomal-dependent degradation of p57 [48]. Therefore, it is reasonable to hypothesize that the increased 1P isoform accumulated upon MG132 treatment might correspond to the above-mentioned phosphorylated forms, or might alternatively be a p57 phosphoisoform whose pI shifts towards more acidic forms when phosphorylated at Ser 282 or Thr 310.

It is well known that p57 and the other CIP/Kip family members exert the critical role of negative regulators of cell proliferation through their capability to associate to different CDKs and inhibit their activity. In view of our finding that p57 is largely phosphorylated, it appeared of interest to characterize the p57 phosphoisoform pattern associated with distinct CDKs. Therefore, endogenous CDK4, CDK6, CDK2, and CDK1 were immunoprecipitated from total Lan-5 protein extracts. Subsequently, the content of p57 isoforms was analyzed by 2D/WB in the specific immunoprecipitated material. In Figure 3F, different isoforms of p57 were found to be associated with distinct CDKs, suggesting a role of phosphorylation in the modulation of CDKs’ binding. Interestingly, the unmodified and monophosphorylated forms of the p57 mostly interact with CDK4. Conversely, the monophosphorylated and the biphosphorylated forms are involved in the binding with CDK6 and CDK2, while the most phosphorylated p57 isoforms are involved in the binding with CDK1, in accordance with the pattern observed by the protein in the G2/M phase. In brief, we demonstrated that the specific phosphorylation of the protein modulates the selective binding with CDKs.

### 2.4. p57 Isoforms Specifically Bind Non-Canonical p57 Partners LIMK1 and CRM1

An increasing body of evidence has demonstrated that p57 has numerous interactors in addition to the cyclin/CDK complexes. Since we observed a different distribution of p57 phosphorylations in the nuclear and cytosolic subcellular compartments, we investigated whether specific isoforms are involved in the binding with a cytoplasmic interactor of p57, like LIMK1 [21]. In addition, since it has been proposed that the cytoplasmic delocalization of p57 might be due to a CRM1-dependent nuclear export [55], and the binding can be promoted by phosphorylation, we investigated whether selective p57 phosphoisoforms are involved in the binding with CRM1. As for CDKs, immunoprecipitation of, respectively, LIMK1 and CRM1 was performed and the immunopurified material analyzed by 2D/WB. Since the amount of p57 interacting with both LIMK1 and CRM1 was not sufficient for performing definite 2D/WB analyses, the co-transfection of both proteins under investigation was performed.

**Figure 4:**
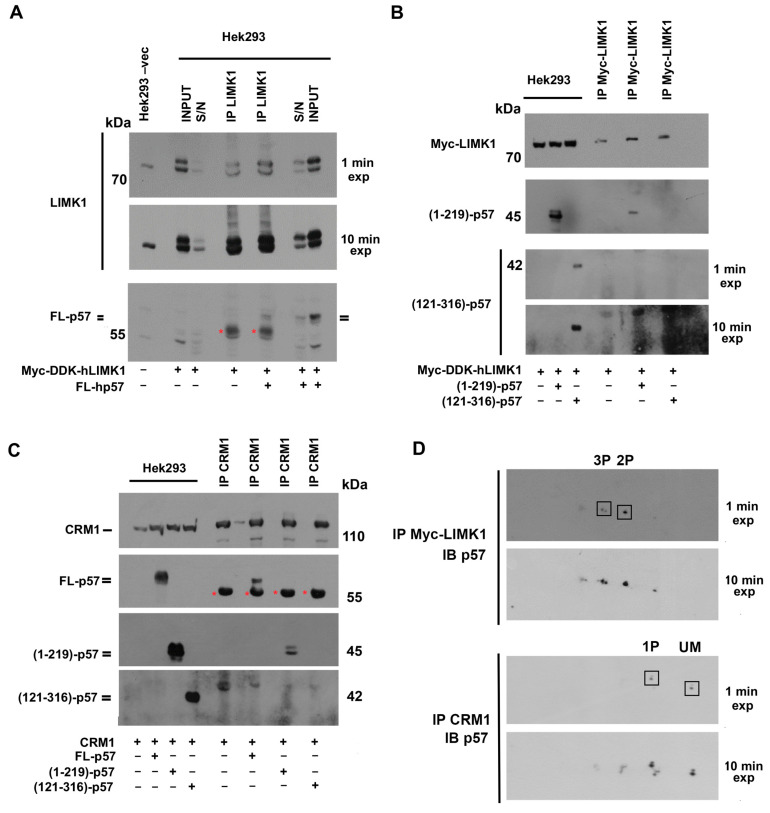
Analysis of p57 isoforms in the association with LIMK1 and CRM1. (**A**) The 1D/WB analysis of p57 content in the immunoprecipitation of LIMK1 from total protein extracts of Hek293 cells co-transfected for 24 h with 1 µg of both p57-FL pcdna3.1 plasmid and Myc-DDK-hLIMK1 pCMV6-Entry plasmid. Hek293 cells transfected with an empty vector were used as a negative control of transfection, and total extract is loaded as a reference for LIMK1 and p57 endogenous signals. Hek293 cells transfected with only Myc-DDK-hLIMK1 pCMV6-Entry plasmid were used as a negative control for the IP experiment. Immunoprecipitation (IP) with a rabbit pAb anti-LIMK1 was performed from both cells co-transfected and transfected only with the Myc-DDK-tagged hLIMK1 plasmid. Total extract (INPUT), LIMK1 IP, and supernatants (S/N) after LIMK1 IP were analyzed for p57 and LIMK1 content in 1D/WB. * in red indicates IgG heavy chain’s signal. (**B**) The 1D/WB analysis of the p57 N-term and C-term fragment content in the immunoprecipitation of Myc-DDK-LIMK1 from total protein extracts of Hek293 cells co-transfected as in panel A except that N-term (1–219)-hp57 and the C-term (121–316)-hp57 pcDNA3.1 plasmids were employed. Immunoprecipitation with a mouse anti-Myc mAb was performed from all three transfected conditions. Total extracts and Myc-LIMK1 IP were analyzed for p57 and Myc-tagged LIMK1 content in 1D/WB. A mouse monoclonal and a rabbit polyclonal Ab were employed to detect, respectively, the N-term (1–219)-hp57 and C-term (121–316)-hp57 fragments. Images acquired at different blot exposure times (1 min and 10 min of exposure) are reported. (**C**) The 1D/WB analysis of p57 content in the immunoprecipitation of CRM1 from total protein extracts of Hek293 cells co-transfected as in panels (**A**,**B**) except that a CRM1 pcDNA3.1 plasmid was employed. Immunoprecipitation with a mouse anti-CRM1 mAb was performed from all four transfected conditions. Total extracts and CRM1 IP were analyzed for p57 and CRM1 content in 1D/WB. A mouse monoclonal anti-p57 Ab was used to detect the FL-p57 and N-term (1–219)-hp57 fragment. A rabbit polyclonal anti-p57 Ab was employed to detect the C-term (121–316)-hp57 fragment. * indicates IgG heavy chain’s signal. (**D**) The 2D/WB analysis of p57 content in the immunoprecipitated material from Myc-LIMK1 and CRM1 immunoprecipitations. Boxes highlight the differences in p57 isoforms involved in the binding with the two interactors analyzed. Specifically, the unmodified (UM) and the monophosphorylated (1P) forms are found in the binding with CRM1, while more acidic (2P and 3P) isoforms are prevalently found in the binding with LIMK1. Images acquired at different blot exposure times (1 min and 10 min of exposure) are reported.

Firstly, p57 isoforms involved in the binding with LIMK1 were evaluated. LIMK1 is a serine/threonine kinase downstream of the RhoA-ROCK pathway, involved in the regulation of actin dynamics, mainly via the phosphorylation and inactivation of the actin-binding protein cofilin. Co-transfection was performed in Hek293 cells by using plasmids containing the CDS of the FL human p57 and Myc-DDK-tagged human LIMK1 (hLIMK1). After 48 h, transfected cells were collected, centrifuged, and lysed. The immunoprecipitation was performed employing an anti-LIMK1 antibody. Immunoprecipitation of the LIMK1 from the protein extract of cells transfected only with the plasmid for Myc-DDK-tagged hLIMK1 (and not with p57) was performed as a control. In Figure 4A, 1D/WB using anti-p57 rabbit Abs revealed the presence of the p57 doublet signal in the LIMK1-immunoprecipitated material only from co-transfected cells. The asterisks mark the heavy chains of the rabbit IgG used for IP. anti-LIMK1 Ab was loaded as a reference for IgG (Figure S4). We also performed experiments using expression vectors for the N-term (1–219)-p57 and the above-described C-term (121–316)-p57 fragments that overlap in the p57 central PAPA region, reported as involved in the interaction with LIMK1. These vectors were used in a co-transfection experiment with the Myc-DDK-tagged hLIMK1 plasmid. The analysis in 1D/WB, reported in Figure 4B, showed that the N-terminal fragment (1–219)-p57, containing the KID and the PAPA region, co-immunoprecipitated with LIMK1. Conversely, the C-terminal fragment (121–316)-p57, also containing the PAPA region, was not detected in the immunoprecipitated material. This result suggests that the integrity of the N-terminal and of the central domain of hp57 and/or the PTMs occurring on these domains may be necessary for the binding with LIMK1.

The association of p57 with CRM1, the major player in nuclear-cytosolic transport, was then evaluated. It has been suggested, although not proved, that this interaction might involve the N-terminal region of p57, as occurs for the other CIP/Kip proteins. The binding should result in the translocation to the cytoplasm. Thus, the association of CRM1 with the FL-hp57 and the N-terminal and C-terminal p57 fragments was investigated. The experiments were essentially performed as those reported for LIMK1. Immunoprecipitation was also carried out employing an extract from cells transfected with only the CRM1 plasmid as a control. As shown in Figure 4C, the analysis of the immunoprecipitated material by 1D/WB demonstrated that FL p57 interacts with CRM1, and that the interaction occurs particularly with the N-terminal (1–219)-p57 fragment.

Subsequently, to characterize the phosphoisoforms involved in the binding, the materials from both LIMK1 and CRM1 immunoprecipitation experiments were analyzed by 2D/WB (Figure 4D). A major finding, emerging from the identification of p57 phosphoisoforms interacting with the two proteins, was that a specificity of the binding exists. In other words, the p57 phosphoisoforms interacting with LIMK1 are mostly different from those interacting with CRM1. In addition, by comparing different film exposures, it was possible to verify that a particular biphosphorylated form of p57 interacts with LIMK1. In the case of CRM1, the most intense monophosphorylated form found in the interaction with CRM1 corresponds to the upper 1P isoform described in Figure 2B. Since we found that this isoform increases as the cycle proceeds from G0 to G1/S, it is reasonable to hypothesize that this phosphorylation contributes to the delocalization of the protein in the cytoplasm as an additional mechanism to ward off and inhibit the function of p57 as an inhibitor of cell cycle progression.

## 3. Discussion

This is, to our knowledge, the first detailed demonstration that a high percentage of cellular p57 content occurs as a phosphoprotein in different cellular models. We showed that phosphorylation represents the main PTM of the protein and that it influences p57 localization, stability, and interaction with CDKs, LIMK1, and CRM1. Moreover, the 2D/WB analysis of the nuclear and cytoplasmic protein extracts allowed us to demonstrate that the unmodified form of the protein localizes only in the nucleus together with the less phosphorylated forms of p57. Conversely, in the cytoplasm, the most phosphorylated forms of p57 are detected. The occurrence of superimposable p57 bidimensional patterns in different cell lines suggests the maintenance of the main phosphorylated isoforms of the protein in different phenotypes. Finally, the pattern of monophosphorylated p57 isoforms indicates the occurrence of at least five different p57 phosphorylatable residues (Figure 1F) even if the complexity of the two-dimensional pattern argues for the possible presence of additional (i.e., more than five) residues target of phosphorylation.

p57 is the least studied CIP/Kip protein and scarce amounts of data have been so far reported in the literature about its metabolism (including its PTMs), clearly showing a gap in the knowledge when compared to p21^CIP1/Waf1^ and p27^Kip1^. In contrast, a growing body of evidence highlighted the multifunctional versatility of p57 that appears to be involved in several biological and pathological processes beyond its role as a CKI [14,41]. These roles include the modulation of the activity of transcription factors [61,62,63,64], cytoskeleton remodeling [21,22,23], cell death [4,24,25], DNA damage and cellular stress response [65,66,67,68], regulation of metabolism [69,70], stem cell fate [64,71,72,73,74], cell differentiation [14,64], and development [14,64]. In cancer development, differently from other CDK inhibitors, the precise role of p57, although evident, is still a matter of debate [3,75,76,77]. Additionally, a function of p57 in response to cancer therapy has been reported [65,78]. p57 belongs to the IUP family, namely, proteins lacking a clearly defined ternary structure [36]. This characteristic allows IUPs to have many interactors permitting transitory but highly specific context-dependent functions. In this scenario, the characterization of the p57 PTMs becomes a primary goal.

Previously, we demonstrated that p57, at least, in the erythroleukemic cell line K562, is post-translationally modified, specifically, it is phosphorylated [60]. In this report, we performed an in-depth characterization of the p57 phosphoisoform pattern by means of bidimensional electrophoresis followed by immunoblotting as a powerful tool for studying proteins [60]. This technique appears to be extremely useful since phosphorylation strongly affects the protein pI, particularly, in our experience, when the isoelectric point of the unmodified protein is in the range between 4 and 8. Accordingly, the calculated pI of the human p57 protein is 5.39, namely in the interval of pIs reported. In all the investigated tumoral and not-tumoral cell lines, including neuroblastoma cells, cervical carcinoma cells, and the extravillous trophoblast cells HTR8/SVneo, the immunoreactive signal of p57 appears in 1D/WB as a doublet. The 2D/WB analysis showed that a large majority of p57 is phosphorylated and, importantly, that numerous distinct residues are modified. The 2D/WB analysis of STS-treated HeLa protein extracts incubated with λ-PPase and the analysis of p57 synthesized in vitro confirmed that in the 2D p57 pattern, the spot that focalizes at about pH 5.4 corresponds to the unmodified form of the protein (referred to as the UM spot) while the other immunoreactive spots correspond to putatively phosphorylated forms of the protein. Of interest, the analysis at a higher resolution of the p57 2D/WB pattern in HeLa cells allowed us to recognize among the monophosphorylated isoforms at least five distinct phosphorylated residues (Figure 1F).

Although the identification of the numerous modified residues is still under development, this study definitely demonstrates that p57 phosphorylation strongly affects the protein functions. First of all, the distribution of different p57 phosphoisoforms in the nucleus and cytoplasm subcellular compartments is clearly distinct. The 2D/WB analysis in Lan-5 cell extracts showed that the p57 unmodified form and the few hypo-phosphorylated forms of the protein are localized in the nucleus, while the hyper-phosphorylated isoforms are detectable mainly in the cytoplasm. This result underlines how phosphorylation strongly influences and probably regulates p57 localization. Previously, we identified an NLS at residues 312-RKRLR-316 (i.e., at the C-end of p57) that allowed us to conclude that p57 presents a bipartite NLS [34]. When we investigated the subcellular distribution of the phosphoisoforms of the C-term (121–316)-p57 fragment, we evidenced that the unmodified form of this p57 C-term fragment and the hypophosphorylated forms of the fragment show a nuclear localization, while the most phosphorylated isoforms localize in the cytoplasm. The evidence supports the hypothesis of a metabolism of the C-term fragment similar to that of the full length p57, and suggests that phosphorylation(s) at the 121–316 residues might be involved in the nuclear-cytoplasm shuttling of the protein. Previously, Zhao and colleagues reported that in Her2-amplified breast cancer cells, phosphorylation at Ser 282 of human p57 by Akt modulates the stability and the cytoplasmic delocalization of the protein, in accordance with our observations [48].

In the context of the nuclear/cytosol shuttling of p57, we report, for the first time, that human p57 is able to bind CRM1, the major exportin protein. So far, only indirect information is available in the literature on the mechanism of p57 nuclear exit. In particular, Gottle and colleagues demonstrated that intracellular protein shuttling is relevant for myelinating glial cells, highlighting the importance of the subcellular localization of p57 in the maturation of oligodendrocytes in mice [55,79]. The authors reported that the translocation of p57 from the nucleus to the cytoplasm contributed to the removal of the p57 inhibitory activity both on cell cycle-related CDKs, and on transcription factors like Mash1. Mash1 is responsible for the differentiation of oligodendrocyte precursors into myelinating mature neuroglial cells. Through an in silico investigation, the authors also identified a putative nuclear export sequence (NES; 43-LGRELRMRLAEL-56) within the KID of p57. Since the NES sequence is required for binding with CRM1, they verified that in oligodendrocyte’s precursors exposed to ratjadone, a selective inhibitor of CRM1, mp57 accumulated in the nucleus and, in parallel, inhibited the differentiation process [55]. Here, we confirm and extend Gottle’s data demonstrating that CRM1 interacts with the human p57, and that the interaction involves the N-terminal 1–219 amino acids, according to the in silico prediction reported for the mp57. Furthermore, we observed that two monophosphorylated forms of the FL-hp57 are involved in the binding with CRM1, thus suggesting that, beyond the phosphorylation on Ser282 reported in breast cancer cells, additional and/or context-dependent molecular phosphorylation might regulate the cytoplasmic-nuclear shuttling of hp57. Further investigations are under development to identify the phosphoisoforms involved in the binding with CRM1 and to clarify their role in the cytoplasmic export of the p57.

Localization, stability, and binding of CIP/Kip proteins with cyclin/CDK complexes can be modulated by phosphorylation through cell cycle phases. Preliminarily, we observed that hp57 is highly expressed, as expected, in starved and G1 phase-synchronized cells, while the content of the protein abruptly decreased in S-phase-arrested Lan-5 cells. In G2 and M phases, the protein increases again and is abundantly phosphorylated, as shown by the characteristic doublet signal appearing in the cytoplasm. The 2D analysis of the protein extracts allowed us to make additional considerations. First, through all the cell cycle phases, the unmodified form of hp57 is constantly detected in the nucleus together with the less-modified isoforms of the protein. In addition, in the cytoplasm, the most phosphorylated forms of the protein are detected. Second, despite the complexity of the 2D patterns, some cell cycle-dependent changes are evident. Specifically, starting from starvation to the G1/S transition, the increasing intensity of a specific monophosphorylated isoform (the upper of the two spots) was observed in the nucleus. Of interest, this phosphoisoform is also present in p57 bound to CRM1.

We also evidenced that differences exist among the p57 phosphoisoforms interacting with distinct CDKs. The less-phosphorylated hp57 isoforms were detected to be associated with CDK2, CDK4, and CDK6. Specifically, unmodified p57 is abundant in CDK4-immunoprecipitated materials. Conversely, the most phosphorylated forms of hp57 were found to be associated with CDK1. Previous mechanistic studies reported that, in in vitro experiments, hp57 inhibits the kinase activity of distinct cyclin-CDK complexes with different effectiveness [1,2,80,81]. More intriguingly, no inhibition of cyclin D-CDK4/6 complexes was found at the equimolar ratio between the CKI and the cyclin/CDK complex. Structural analyses revealed differences in the organization of the ternary complex, compared to that formed with CDK2 [82,83]. In particular, LaBear and colleagues in 1997 demonstrated that CIP/Kip proteins, including p57, facilitate the activation of cyclin D-CDK4/6 carrying them into the nucleus, in a dose-dependent manner. Our observation that different hp57 phosphoisoforms are bound to distinct cyclin/CDK complexes might help in explaining the different effects of p57 on the kinase activity. In other words, a different phosphorylation of p57 might be involved, at least in part, in a distinct interaction/effect on the cyclin/CDK complex. In addition, we propose that shedding light on the phosphorylation pattern of p57 is essential not only for the comprehension of the functions of this protein, but also to better understand its putative role in the response to cancer therapies. The finding is of peculiar relevance in view of the observation that a specific monophosphorylated form of hp57 is involved in the binding with CDK4. Of note, palbociclib, a selective CDK4/6 inhibitor (CDK4/6i), in combination with endocrine therapy, represents the first-line treatment for metastatic postmenopausal patients with estrogen receptor-positive and Her2 (Human epidermal growth factor receptor 2)-negative breast cancer [84]. Several CDK4/6i analogs have been developed and are in clinical trials. However, although CDK4/6i analogs have made a significant therapeutic progress not only in the treatment of breast cancer, their clinical efficacy is still limited by drug resistance occurrence. Recently, it has been shown that acquired resistance to palbociclib may arise from activated non-receptor tyrosine kinases that phosphorylate and “activate” p27^Kip1^ [59], promoting the assembly of a trimeric pYp27-CDK4-cyclin D1 complex that favors the binding of ATP to the kinase, enhancing its activity. On these bases, the complete identification and comprehension of the role of p57 phosphoisoforms interacting with CDK4/6 could be of interest for targeted therapy response.

CDK1 is an essential CDK whose ablation leads to the arrest of embryonic development [1]. Since we observed that the most phosphorylated isoforms of hp57 interact with CDK1, we hypothesized that the role of the protein in the CDK1 activity modulation at the G2/M transition of the cell cycle could be related to the high phosphorylation state of the protein. It has been reported that mp57 can bind and inhibit CDK1 to prevent placental trophoblast stem cells from entering mitosis and promote endoreplication with consequent differentiation into trophoblast giant cells. These events have been described to occur in a coordinated action with p21 with the aim of inhibiting apoptosis [85]. In addition, other authors suggested that mouse chondrocyte differentiation can be promoted through the p57 inhibition of CDK1. Accordingly, the knowledge of the molecular mechanism of this activity might be important for the development of therapeutic strategies for the treatment of osteoarthritis and osteochondrodysplasias [86,87]. Thus, the comprehension of the effect of p57 phosphorylation(s) on the activity of CDK1 will be of interest in explaining the involvement of p57 in the aforementioned differentiating processes, with the aim of developing targeted therapies for various pathologies. As matter of fact, there is a significant amount of the literature on the involvement of p57 in differentiation processes of various tissues, but little is known regarding the role of p57 PTMs in their modulation [14,64].

In addition to the activity exerted on CDKs, cytoplasmic p57 functional partners have also been reported, including LIMK1. In more detail, it has been reported that the central region of hp57, the PAPA region, can be involved in cytoplasmic interactions regarding actin cytoskeleton dynamics and apoptosis [21,88]. Particularly, the PAPA region of hp57 interacts with LIMK1, a Ser/Thr kinase downstream from the Rho/ROCK pathway, that phosphorylates on Ser3 the ADF-family protein cofilin, inhibiting actin severing. This, in turn, prevents cell migration and invasion [21,88]. In the same context, it has been observed that the p57-mediated activation of LIMK1 can also promote mitochondrial-induced apoptosis through the stabilization of the actin fibers and the hexokinase 1 dislocation from the mitochondria [25]. However, the differences in the modulation of LIMK1 activity between mouse and human proteins are still unclear. Indeed, if some authors reported that mp57 inhibits LIMK1 activity by delocalizing and trapping LIMK1 in the nucleus [55,88], others described that, in tumor cells, hp57 promoted the cytoplasmic activity of LIMK1 [22,25,61,89,90,91]. Whether the modulation of actin dynamics is cell-context dependent or involves additional LIMK1 effectors is not known and needs further investigation in non-tumoral cells, especially for hp57. Here, we showed that some highly phosphorylated forms of hp57, not found to be involved in the binding with CDKs and CRM1, interact with LIMK1.

Finally, due to the relevance of p57 in multiple physiological and pathological processes, it is mandatory in the next future to precisely identify the p57 phosphorylated residues in order to unravel the functions, still not completely understood, of this CIP/Kip protein, especially in humans. In this study, we have demonstrated how a two-dimensional technique can be useful in the recognition of the phosphoisoforms of p57. The combination of site-directed mutagenesis (to abrogate putative phosphorylatable residues or to obtain phosphomimetics) and the subsequent 2D/WB analysis would represent an effective strategy for the comprehension of the p57 2D/WB pattern. Moreover, mass spectrometry and the production of p57-specific anti-phosphoresidue antibodies would allow a definite confirmation of the identified phosphoisoforms. Mechanistic studies will finally be needed to unravel their function in physiological and pathological conditions.

## 4. Materials and Methods

### 4.1. Cell Culture and Treatments

All the cell lines employed were cultured following ATCC (ATCC, Manassas, VA, USA) instructions. Specifically, the human neuroblastoma cell lines LAN-5, SH-SY5Y and SK-N-BE cell lines were grown in 100 cm^2^ tissue culture dishes with RPMI (Gibco, Thermo Fisher Scientific, Waltham, MA, USA), supplemented with 10% fetal bovine serum (FBS, Thermo Fisher Scientific), 100 U/mL of benzylpenicillin, 100 mg/L of streptomycin (Gibco), in a humidified incubator with 5% CO_2_ at 37 °C. The human trophoblast cell line HTR8/SVneo was grown as described above except that it was in RPMI medium supplemented with 5% FBS. The human cervical cancer cell line HeLa, the osteosarcoma cell line U2OS, and the human embryonic kidney 293 cells (Hek293) were grown as described above except that they were in DMEM medium. To increase p57 expression, 100 nM of Dexamethasone (Sigma-Aldrich, St. Louis, MO, USA) was added to the culture medium for 24 h. The proteasome inhibitor MG132 (Sigma-Aldrich) was used to prevent the proteasomal degradation of p57 at the concentration of 10 µM in the culture medium for 5 h. Thymidine (Sigma-Aldrich) and nocodazole (Sigma-Aldrich) were used to arrest cells, respectively, in the S- and M-phases. Staurosporine (Sigma-Aldrich) was employed as a broad-spectrum kinase inhibitor. All the reagents were of the highest purity grade commercially available.

### 4.2. Cell Synchronization and Verification by FACS

For cell cycle analysis, Lan-5 cells were synchronized as follows. Cells were starved by plating them at a confluency of 30–40% and incubated in serum-free fresh medium for 72 h (G0 phase). After starvation, the serum-free medium was removed, and the cells were maintained for 8 h (G1 phase) and 12 h (G1/S transition) with 10% FBS-containing medium, allowing them to re-enter the cell cycle, and then collected for lysis. To block cells in the S phase, 2 mM of deoxythymidine was added to the medium, and cells were collected after 48 h. Following thymidine treatment, the thymidine-containing medium was removed, Lan-5 cells were washed, and then a fresh medium was added to release cells. After 16 h, adherent cells were collected (G2 phase). To select cells arrested in the M phase, 10 nM of nocodazole was added to the medium for 16 h and then rounded-up cells were separated from those firmly attached to the culture substrate through the gentle shaking of the culture dish, thus allowing the detachment of the mitotic cells.

Cell synchronization was evaluated by FACS analysis as previously described [92]. Briefly, cells were collected, washed twice in phosphate buffer (PBS), and centrifuged at 800× *g* for 5 min. Subsequently, the cellular pellets were incubated in a lysis solution containing 2 mM of propidium iodide, DNase-free RNase (Sigma) at RT for 30 min, and then analyzed by flow cytometry using a FACSCalibur (Becton Dickinson, Franklin Lakes, NJ, USA). Cell cycle distribution was calculated from 60,000 events with ModFit LTTM vers. 3 software (Becton Dickinson).

### 4.3. Cell Extract Preparation and Mono- and Two-Dimensional Western Blotting Analyses

Protein extracts were prepared as previously described in [93]. Particularly, for whole-cell extract preparation, cells were lysed in ice-cold RIPA buffer supplemented with protease and phosphatase inhibitors. Nuclear and cytosolic fractionation was performed, resuspending cells in a chilled hypotonic buffer for 20 min on ice, allowing cells to swell. Subsequently, IGEPAL CA-630 solution (Sigma) was added for 15 s to a 0.6% final concentration, and then cells were centrifuged at 10,000× *g* for 2 min. The cytosolic fraction was recovered, and the pellet nuclei were then lysed in RIPA buffer for 30 min on ice. Nuclear and cytoplasmic extracts obtained were tested for cross-contamination through the 1D/WB of Lamin A/C or HDAC1 and LDH, respectively.

One-dimensional SDS-PAGE was used to separate proteins based on their molecular weight. Two-dimensional SDS-PAGE was used to analyze proteins based on their isoelectric point and molecular weight. The 1D/WB and 2D/WB were performed as previously reported [67,94]. The theoretical isoelectric point of hp57 and of the C-term (121–316)-p57 fragment were calculated using the program ExPASy Proteomics Tools (https://web.expasy.org/compute_pi/, accessed on 22 September 2024). Accordingly, Immobiline DryStrip gels, linear pH 4–7 and 3–10 (Cytiva, Marlborough, MA, USA) have been employed for the FL-p57 and the C-term fragment. Isoelectrofocusing and 1D SDS-PAGE were performed as previously reported [67,94]. The following primary antibodies were employed: anti-p57 (mouse monoclonal), anti-Lamin A/C (mouse monoclonal), anti-LDH (mouse monoclonal), anti-CDK1 (mouse monoclonal), anti-CDK4 (rabbit polyclonal), anti.CDK6 (rabbit polyclonal), anti-CDK2 (mouse monoclonal), anti-HDAC1 (mouse monoclonal), anti-CRM1 (mouse monoclonal), and anti-cMyc (mouse monoclonal), all purchased from Santa Cruz Biotechnology (Santa Cruz Biotechnology, Inc., Heidelberg, Germany); anti-actin (rabbit polyclonal) and anti-p57^Kip2^ (rabbit polyclonal), purchased from Sigma-Aldrich; and anti-LIMK1 (rabbit polyclonal) from Cell Signaling Technology (Leiden, The Netherlands). HRP-conjugated secondary antibodies (Jackson ImmunoResearch Europe LTD, Cambridgeshire, UK) were applied to bind to and visualize primary antibodies.

### 4.4. Phosphatase Assay and IVTT Reaction

For the phosphatase assay, the lambda protein phosphatase (λ-PPase) enzyme (Santa Cruz Biotechnologies), a protein phosphatase capable of removing phosphate from residues of phosphorylated serine, threonine and tyrosine, was used. Particularly, 100 units of λ PPase removed 250 picomoles of phosphate in 30 min at 30 °C. Whole HeLa cell extracts and the recombinant hp57, prepared and immunopurified from an in vitro transcription and translation reaction (IVTT), were used as substrate for the phosphatase assay. The kit TnT Quick Coupled Transcription/Translation Systems used for the IVTT were provided by Promega Corporation (Madison, WI, USA). The IVTT reaction was diluted in RIPA buffer and recombinant hp57 was immunoprecipitated with a rabbit polyclonal anti-p57 Ab conjugated to protein A/G Agarose beads (Santa Cruz Biotechnology, Inc., Heidelberg, Germany). Recombinant hp57 bound-agarose beads were washed twice with RIPA buffer (not supplemented with PPase inhibitors), and once with the PPase reaction buffer (i.e., 50 mM Tris-HCl, 0.1 mM Na_2_EDTA, 5 mM DTT, 0.1% lauryl ether Brij-35, 2 mM MnCl_2_). Subsequently, after drying the recombinant hp57-beads, the immunoprecipitated protein was incubated for 1 h at 30 °C in 50 µL of reaction buffer with 400 units of the enzyme λ-PPase. At the end of the reaction, the hp57-beads were centrifuged at 4000× *g* and the supernatant was discarded. Then, the immunoprecipitated and dephosphorylated protein was detached from agarose beads with 100 mM of Glycine-HCl, pH 2.3, and subsequently precipitated with 10% TCA, as reported above. The obtained pellet was resuspended in urea-containing buffer for 2D analysis.

### 4.5. Immunoprecipitation

Immunoprecipitation was performed by using protein A/G agarose plus (Santa Cruz Biotechnology, Inc.), according to the manufacturer’s instructions. Different amounts of protein extracts were employed and incubated overnight with 2–3 µg of a specific antibody on a wheel at 4 °C. Subsequently, the immuno-complexes were transferred on the immunoaffinity matrix and incubated 2 h on wheel at 4 °C, allowing the binding of the immuno-complexes to the protein A/G. Finally, the immunoprecipitated material was eluted in a loading sample buffer for 1D/WB analysis or processed as follows for 2D analysis. The beads were washed three times with PBS and then incubated with Glycine-HCl, allowing the elution of the bound immunopurified material. In particular, 300 µL of 100 mM of Glycine-HCl, pH 2.3, were added to dried packed beads and incubated at room temperature for 20 min on a gentle shaker. After centrifugation (1200× *g*, 5 min), the elution containing the immunopurified material were recovered. The pH of the eluates was immediately adjusted to 7.0 using 1 M of Tris/HCl, pH 8.5.

### 4.6. Plasmid Transient Transfection

hp57-FL, the N-term (1–219)-hp57, and the C-term (121–316)-hp57 plasmid preparation and transfection were performed as previously [34]. The coding sequence of CRM1 was cloned in the pcDNA3.1 plasmid. Each cloning product was confirmed by direct sequencing [95]. The Myc-DDK-LIMK1 pCMV6-Entry plasmid was purchased from Origene (OriGene Technologies, Inc., Rockville, MD, USA). The 60% confluent Hek293 cells were transfected with 1–2 µg of plasmid using jet PRIME (Polyplus Now Part of Sartorius, Illkirch, France), according to the manufacturer’s instructions. After 24 h, cells were processed for subsequent analyses.

## 5. Conclusions

The analysis of the human p57 phosphorylations reported in this study is undoubtedly unique and has not been previously described. We have shown that phosphorylation represents the main PTM of the hp57, recognizing at least five monophosphorylated isoforms by 2D/WB analysis. Indeed, compared to other CIP/Kip proteins, hp57 appears to be abundantly phosphorylated in tumoral and not-tumoral cell models, especially in the cytoplasm of synchronous and asynchronous analyzed cells. Moreover, different phosphoisoforms of the protein have been found in the interaction with the canonical functional partner CDKs and with the non-conventional interactors LIMK1 and CRM1. It is a matter of fact that phosphorylation influences the stability, localization, interactors, and functions of IUP proteins like CIP/Kip. However, so far, little is known regarding the post-translational modulation of the hp57 compared to its siblings. Phosphorylations at serine 282 and threonine 310 have been reported to modulate the stability and localization of the protein in breast cancer cells [48], but these are not sufficient to explain the complexity of the hp57 2D pattern. Thus, it is mandatory to identify all the phosphorylated residues of hp57 and the kinases/pathways responsible for these modifications. This will allow us to fully unravel the roles of this multifunctional protein, which are still only partially identified. Furthermore, it will also be of great importance in characterizing the molecular mechanisms of the sensitivity/resistance to targeted cancer therapy.

## Data Availability

Data are contained within the article and Supplementary Materials.

## Supplementary Materials

The following supporting information can be downloaded at: https://www.mdpi.com/article/10.3390/ijms252011176/s1.
