# Supplementary material for: p57Kip2 Phosphorylation Modulates Its Localization, Stability, and Interactions
Source: Int J Mol Sci. 2024 Oct 17;25(20):11176. doi: 10.3390/ijms252011176 (PMC11508627; doi:10.3390/ijms252011176)
Supplement: Supplementary file 1 [file ijms-25-11176-s001.zip › ijms-3249062-supplementary.pdf]

Supplementary Materials

$p57^{Kip2}$ Phosphorylation Modulates Its Localization, Stability, and Interactions

Emanuela Stampone [1,*,†], Debora Bencivenga [1,†], Luisa Dassi [1], Sara Sarnelli [1], Luisa Campagnolo [2], Valentina Lacconi [2], Fulvio Della Ragione [1] and Adriana Borriello [1,*]

[1] Department of Precision Medicine, University of Campania "Luigi Vanvitelli", 80138 Naples, Italy; debora.bencivenga@unicampania.it (D.B.); luisa.dassi@unicampania.it (L.D.); sara.sarnelli@unicampania.it (S.S.); fulvio.dellaragione@unicampania.it (F.D.R.)

[2] Department of Biomedicine and Prevention, University of Rome Tor Vergata, 00133 Rome, Italy; valentina.lacconi@uniroma2.it (V.L.)

* Correspondence: emanuela.stampone@unicampania.it (E.S.); adriana.borriello@unicampania.it (A.B.); Tel.: +39-081-566-7545 (E.S.); +39-081-566-7554 (A.B.)

† These authors contributed equally to this work.

Table of Contents	Page
A. Supplemental Materials and Methods	3
Cell culture and treatments	3
Immunoblotting	3
B. Supplemental Figures	
Figure S1 Analysis of FL-p57 2D pattern overexpressed in Hek293 and U2OS cell lines.	4
Figure S2 Analysis of p57 isoforms in Lan-5 differentiated with ATRA and TPA.	5
Figure S3 Analysis of p57 isoforms in HeLa cells treated with 50 μM H_2O_2.	6
Figure S4 Co-immunoprecipitation of FL-p57 with LIMK1.	7

Supplemental Materials and Methods

Cell culture and treatments

The neuroblastoma cell line Lan-5 (ATCC, Manassas, VA, USA) was cultured in RPMI (Gibco, Thermo Fisher Scientific, Waltham, MA, USA). The human cervical cancer cell line HeLa, the human embryonic kidney 293 cells (Hek293), and the osteosarcoma cell line U2OS were grown in DMEM high glucose (Gibco). All cell media were supplemented with 10% fetal bovine serum (FBS, Invitrogen, Thermo Fisher Scientific Waltham, Massachusetts, U.S.A), 100 U/mL benzylpenicillin, and 100 mg/L streptomycin (Gibco) as described in the main text. Lan-5 cells were differentiated by mildly starving cells (1% FBS) for 24 h and subsequently by treating them with 10 μM all-trans retinoic acid (ATRA, Sigma) and 50 nM 12-O-tetradecanoylphorbol-13-acetate (TPA, Cell Signaling) for three days. To induce oxidative stress, HeLa cells were treated with 50 μM H_2O_2 (PanReac Applichem, ITW Reagents, Germany) for 24 h. Subsequently, differentiated Lan-5 cells and stressed HeLa cells were collected and lysed for 2D/WB analysis. Overexpression of FL-p57 was performed in Hek293 and U2OS as described in the main text.

Immunoblotting

1D/WB and 2D/WB were performed as reported in the main text. Accordingly, Immobiline DryStrip gels, linear pH 4-7 (Cytiva, Marlborough, MA, USA) have been employed for the FL-p57. The following primary antibodies were employed: anti-p57Kip2 (rabbit polyclonal) was purchased from Sigma-Aldrich; anti-LIMK1 (rabbit polyclonal) was purchased from Cell Signaling Technology (Leiden, The Netherlands). HRP-conjugated anti-rabbit secondary antibodies (Jackson ImmunoResearch Europe LTD, Cambridgeshire, United Kingdom) were employed to bind to and visualize primary antibodies.

Supplemental Figures

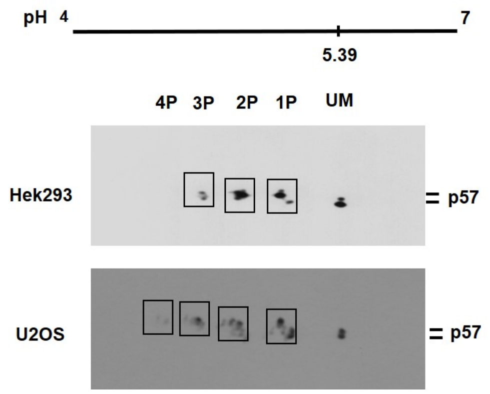

Figure S1. Analysis of overexpressed FL-p57 2D pattern in Hek293 and U2OS cell lines. 60% confluent Hek293 and U2OS were transfected with 1 μg of pcDNA3.1 plasmid containing the coding sequence of FL-p57 for 24 h using Polyplus jet PRIME reagents (Polyplus Now Part of Sartorius, France), according to the manufacturer's instructions. Subsequently, cells were collected and processed for 2D/WB analysis. The two-dimensional pattern of FL-p57 presents multiple phosphorylations in both cell lines. p57 isoforms that focus at the same pH are grouped in boxes like in Figure 1E. The isoform that focuses at pH 5.39 corresponds to the unmodified form of p57 (UM), while the more acidic isoforms correspond to progressively phosphorylated p57 (1P, 2P, 3P and 4P).

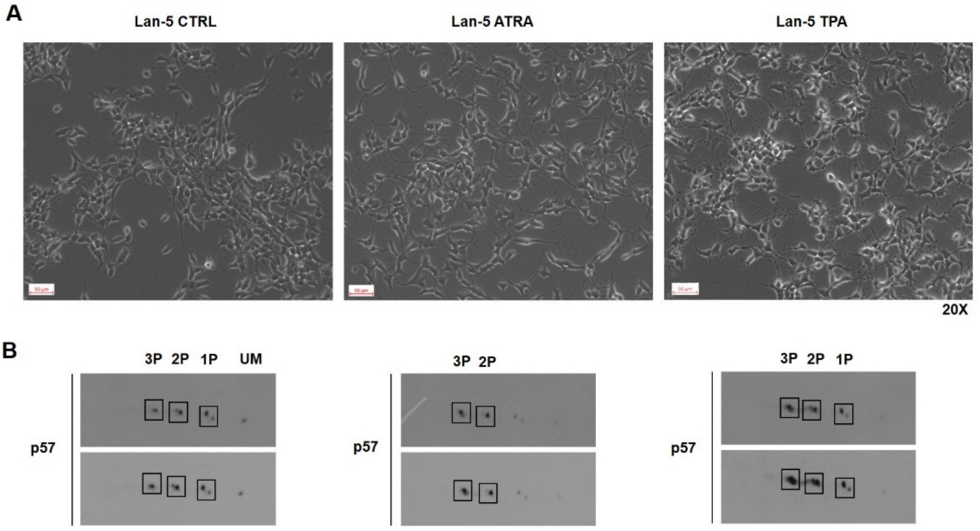

Figure S2. Analysis of p57 isoforms in Lan-5 differentiated with ATRA and TPA. (**A**) Lan-5 cells were mildly starved for 24 h and induced to differentiate through exposure to 10 μM all-trans retinoic acid (ATRA) or 50 nM 12-O-tetradecanoylphorbol-13-acetate (TPA) for three days. Cell images were acquired by light microscopy (Axiovert 5 inverted light microscope, Zeiss) with 20X objective. (**B**) 2D/WB analysis of p57 content in total protein extracts of Lan-5 cells control (CTRL) and treated with ATRA and TPA respectively. Bi-phosphorylated and three-phosphorylated p57 forms (2P and 3P) accumulates when Lan-5 cells were differentiated with ATRA, while increased phosphorylation level of p57 occurred when treated with TPA compared to control.

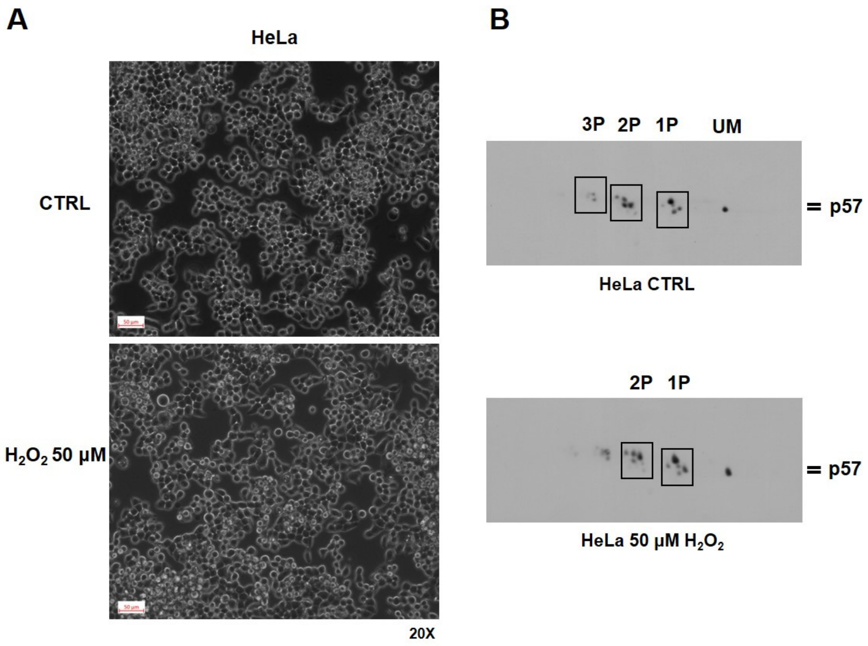

Figure S3. Analysis of p57 isoforms in HeLa cells treated with 50 μM H_2O_2. (**A**) HeLa cells were treated with 50 μM H_2O_2 for 24 h to induce oxidative stress and analyse the effect on p57 phosphorylation pattern compared to control cells. Images were acquired by light microscopy (Axiovert 5 inverted light microscope, Zeiss) with 20X objective. (**B**) 2D/WB analysis of p57 content in total protein extracts of HeLa cells control (CTRL) and treated with H_2O_2. The treatment determines a change in the relative abundances of monophosphorylated (1P) and biphosphorylated (2P) forms of p57.

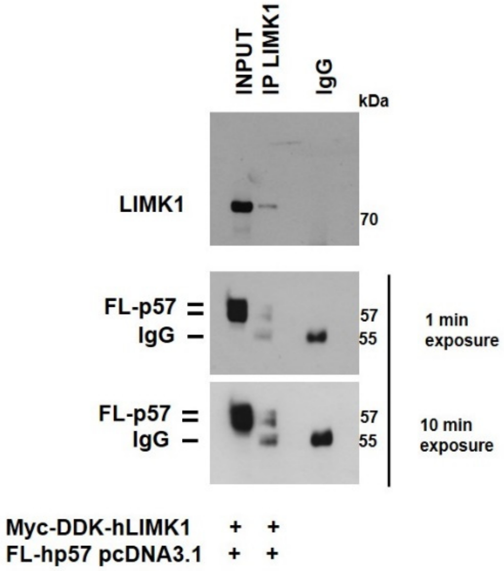

Figure S4. Co-immunoprecipitation of FL-p57 with LIMK1. 1D/WB analysis of p57 content in the immunoprecipitation of LIMK1 from total protein extracts of Hek293 cells cotransfected for 24 h with 1 μg of both p57-FL pcdna3.1 plasmid and Myc-DDK-hLIMK1 pCMV6-Entry plasmid. 500 ng of Anti-LIMK1 rabbit polyclonal Ab were loaded as reference for IgG. Western blotting for FL-p57 was performed with a rabbit polyclonal anti-p57 antibody, same species of anti-LIMK1 Ab, to evidenciate the different migration speed compared to IgG heavy chain, and, thus, the specificity of the p57 signal in the immunoprecipitated material (IP LIMK1). p57 migrates at a higher molecular weight (57 kDa) compared to heavy anti-LIMK1 Ab chains (55 kDa).
